# Increasing temperature weakens the positive effect of genetic diversity on population growth

**DOI:** 10.1002/ece3.8335

**Published:** 2021-12-14

**Authors:** Alexandra L. Singleton, Megan H. Liu, Samantha Votzke, Andrea Yammine, Jean P. Gibert

**Affiliations:** ^1^ Department of Biology Duke University Durham North Carolina USA

**Keywords:** genetic variability, global warming, intraspecific variability, intraspecific variation, microbes

## Abstract

Genetic diversity and temperature increases associated with global climate change are known to independently influence population growth and extinction risk. Whether increasing temperature may influence the effect of genetic diversity on population growth, however, is not known. We address this issue in the model protist system *Tetrahymena thermophila*. We test the hypothesis that at temperatures closer to the species’ thermal optimum (i.e., the temperature at which population growth is maximal, or *T*_opt_), genetic diversity should have a weaker effect on population growth compared to temperatures away from the thermal optimum. To do so, we grew populations of *T*. *thermophila* with varying levels of genetic diversity at increasingly warmer temperatures and quantified their intrinsic population growth rate, *r*. We found that genetic diversity increases population growth at cooler temperatures, but that as temperature increases, this effect weakens. We also show that a combination of changes in the amount of expressed genetic diversity (G) in *r*, plastic changes in population growth across temperatures (E), and strong G × E interactions underlie this temperature effect. Our results uncover important but largely overlooked temperature effects that have implications for the management of small populations with depauperate genetic stocks in an increasingly warming world.

## Graphical abstract

Understanding how temperature influences population growth is important in a warming world. Here, we show that temperature weakens the positive effect of genetic diversity on population growth in a microbial system. These results have consequences for conservation of genetically depauperate populations in a changing world.

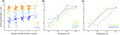

## 1 INTRODUCTION

Rapid global climate change has a myriad of ecological consequences, from individuals to ecosystems (Barbour & Gibert, 2021; Barnett et al., 2005; Bellard et al., 2012; Freeman et al., 2018; Gibert, 2019; Gibert et al., 2016; Gibert & DeLong, 2014; Pimm, 2009). Rising temperatures, in particular, influence metabolic rates (Brown et al., 2004; Gillooly et al., 2002), which determine reproduction (Savage et al., 2004; Schaper et al., 2012; Zeh et al., 2012) and mortality (Amarasekare & Coutinho, 2013; Amarasekare & Savage, 2012), thus setting demographics and population growth (Kremer et al., 2017; Savage et al., 2004). As a consequence, species have thermal tolerances, and these thermal tolerances ultimately determine where on the globe—and under what environmental conditions—species may survive and reproduce (Sunday, Bates, & Dulvy, 2011, 2012). As temperatures increase globally, whether species will shift their geographic ranges (Sunday et al., 2012), or instead go extinct (Freeman et al., 2018), will be largely determined by these temperature tolerances (Calosi et al., 2008).

Genetic diversity has long been known to reduce species extinction risk (Frankham, 2005). For example, genetic diversity is negatively related to extinction risk in birds (Evans & Sheldon, 2008), low genetic diversity increases extinction risk in butterflies (Saccheri et al., 1998), while genetic rescue (i.e., introduction of new genetic variants) decreases extinction risk in mice (Schwartz & Mills, 2005) and pigmy possums (Weeks et al., 2017). Genetic diversity thus hedges against changing environmental conditions by increasing the chance that a population will have individuals with high survival rates in novel environmental conditions. However, a combination of habitat fragmentation and shifting environmental conditions often leads to geographic range reductions (e.g., mountaintop species; Freeman et al., 2018), or crashes in population size (van de Pol et al., 2017). Smaller population size or geographic range strengthens drift and reduces genetic diversity, leading to higher inbreeding depression and extinction risk (Frankham, 2005). Environmentally induced increasing geographic overlap between locally adapted neighboring populations may also increase outbreeding depression, which also has negative consequences for population growth (Frankham, 2005). While both genetic diversity and temperature are well‐known to independently influence population growth (Brown et al., 2004; Frankham, 2005), whether increasing temperatures may alter the effect of genetic diversity on population growth and extinction risk is largely unknown.

Here, we address this issue in a model microbial system, the globally distributed protist *Tetrahymena thermophila*. These organisms play an important role in the global carbon cycle that ultimately determines the pace of climate change (i.e., the microbial loop; Gao et al., 2019; Karhu et al., 2014; Rocca et al., 2021) and are easy to grow in temperature‐controlled laboratory conditions (Fjerdingstad et al., 2007) making them an ideal system to understand how temperature may influence ecological processes (Petchey et al., 1999; Wieczynski et al., 2021).

**FIGURE 1:**
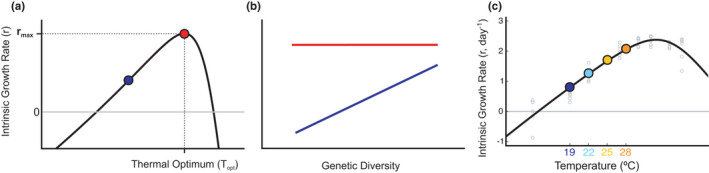
(a) Typical thermal performance curve for the population intrinsic growth rate, *r*, in solid black. Grey solid line represents r=0: above the line, the population grows, below, it decreases. (b) We hypothesize that at temperatures away (blue dot, fig 1a) from the optimal temperature (*T*_opt_, red dot, fig 2a), increasing genetic diversity should lead to increasing intrinsic growth rate (b, blue solid line), while closer to the thermal optimum, increasing genetic diversity should not significantly increase *r* owing to similar growth rates across genotypes (b, red solid line). (c) *Tetrahymena thermophila* thermal performance curve (black line, estimated from real data, in grey). Colored dots indicate experimental temperatures (19ºC, deep blue, 22ºC, sky blue, 25ºC, yellow, and 28ºC, orange)

In particular, we address (1) whether genetic diversity affects population growth in *T*. *thermophila*, (2) whether temperature influences that effect, and 3) through what mechanisms. We hypothesize that lower genetic diversity may depress population growth (lower intrinsic growth rate, *r*), as observed in many other organisms, while higher genetic diversity may increase growth (Frankham, 2005). We also hypothesize that the effect of genetic diversity should be weaker near the species‐level thermal optimum owing to a combination of physiological constraints and differences in how different genotypes grow across temperatures (*T*_opt_, Figure 1a, b): at *T*_opt_, most genotypes should reproduce relatively well, while away from *T*_opt_, some genotypes may perform increasingly poorly. Consequently, increasing genetic diversity may increase the chance of the population having genotypes that reproduce well at temperatures away from *T*_opt_, resulting in a higher intrinsic growth rate with increasing genetic diversity (Figure 1a, b; blue). Conversely, only a weak relationship between genetic diversity and *r*, if any at all, should be observed near or at *T*_opt_ (Figure 1a, b; red). Another interpretation is that physiological constraints on population growth are likely strong at or near *T*_opt_, so that no matter how much genetic variation there is in the population, increasing amounts of genetic variation will not increase growth as much as when those constraints are weaker (e.g., away from *T*_opt_).

## 2 METHODS

### 2.1 Experimental procedure

We sourced five clonal lines (B2086.2, A*III, CU438.1, A*V, and CU427.4) of the protist *Tetrahymena thermophila* from the Cornell University Tetrahymena Stock Center from across three putatively different genetic backgrounds (A*III and A*V have genetic background A, B2086.2 has background B, while CU438.1 and CU427.4 have background C). The lines were reared in Carolina Biological protist medium^®^ (Burlington, NC) in 200 ml autoclaved borosilicate jars, and a 16–8 day/night cycle at 22ºC within Percival growth chambers (Perry, IA).

To determine whether temperature alters the effects of genetic diversity on population growth, we manipulated the temperature and initial genetic diversity of microcosm populations. To manipulate genetic diversity, we started populations with a varying number of clonal lines (1, 2, 3, 4, or 5 lines). Monoclonal cultures were initialized with 50 individual protists. For all other treatments, the initial abundance of each clone depended on the total number of clones present, to control for possible effects of initial density: two‐clone populations started with 25 individuals/clone, three‐clone populations started with ~16 individuals/clone, four‐clone populations started with ~12 individuals/clone, and five‐clone populations started with 10 individuals/clone. Each monoclonal population, and each combination of four and five clones, was replicated four times. Each combination of two and three clones was replicated twice, for a total of 84 experimental populations per temperature. All experimental microcosms were reared in 3 ml of growth media in 35 mm petri dishes.

We crossed the five genetic diversity treatments with four temperature treatments (19, 22, 25, and 28ºC) along the rising portion of the thermal performance curve (TPC) of the organism (Figure 1c), for a total 336 experimental microcosms. The TPC itself was estimated as the intrinsic growth rate, *r*, of a well‐mixed population (i.e., comprising the same number of initial individuals per clone, all starting at 3 ind/ml in 3 ml petri dishes), at seven different temperatures (13, 19, 22, 27, 30, 32, 35, and 37ºC). Experimental microcosms were grown in Percival growth chambers with all other environmental variables mimicking rearing conditions.

After a 24‐h incubation period, we estimated final population size by sub‐sampling each microcosm and counting individual cells under a stereomicroscope (Leica, M205 C). Assuming exponential growth, the intrinsic growth rate (*r*, which has units of *t* ^−1^) of each microcosm population was calculated as [log(*N_f_*)–log(*N_i_*)]/time, with time = 1 day, *N_f_* being the final abundance, and *N_i_* the initial density (=50 ind for all experimental microcosms).

### 2.2 Statistical analyses

We used a linear model with *r* as the response variable, and the number of clones, temperature, and their interaction, as explanatory variables. To understand the mechanisms behind possible effects of temperature on the relationship between genetic diversity and *r*, we assessed whether changes in total additive genetic variation in *r* (G), environmental variation in *r* (E), or G × E interactions, could explain observed changes in *r* across genetic diversity and temperature treatments. To do so, we used Analysis of Covariance (ANCOVA) in R packages “rstatix” v0.7 (Kassambara, 2021) and “emmeans” v.1.6.3 (Lenth, 2021) on data from the monoclonal populations, with *r* as the response variable, and temperature, clonal line, and their interaction, as explanatory variables. We further investigated whether any one clone was responsible for driving some of these patterns by performing a post hoc Tukey test. Last, we tested whether differences in genetic background influenced observed changes in growth rates across temperatures by assessing whether clones with genetic backgrounds A, B, or C grew differently across temperatures. All analyses were done in R v4.1 (R Core Team, 2013). All data and code are publicly available in Dryad (Singleton et al., 2021) or at https://github.com/JPGibert/Genetic_Diversity_And_Temperature.

## 3 RESULTS

**FIGURE 2:**
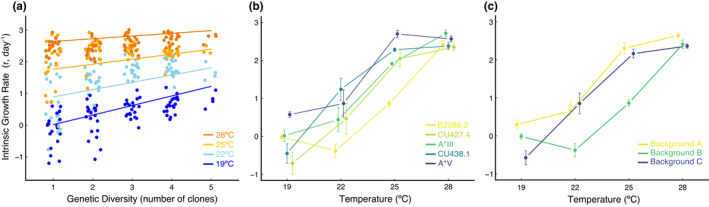
(a) Plot of the intrinsic growth rate, *r*, against the number of clones for all 336 experimental microcosms (dots) across all four experimental temperatures (horizontal *jitter* added for visualization purposes). Solid lines represent linear model predictions (color code as in Figure 1c). (b) Intrinsic growth rate against temperature for all monoclonal cultures. Bars represent standard errors. Lines connecting dots help visualize changes in *r* across temperatures (B2086.2 is yellow, CU427.4 is yellow green, A*III is green, CU438.1 is blue green, and A*V is blue). (c) As in (b) but for strains belonging to the three genetic backgrounds (yellow: A, green: B, and blue: C)

Population intrinsic growth rate (*r*) increased with temperature (estimate = 0.32 ± 0.02, *t* = 17.73, *p* < .001, Figure 2a) and with genetic diversity (estimate = 0.76 ± 0.15, *t* = 5.17, *p* <.001, Figure 2a) as hypothesized. The positive effect of genetic diversity on *r* decreased with temperature (estimate = −0.02 ± 0.006, *t* = −3.89, *p* < .001, Figure 2a), also in accordance with our hypothesis.

ANCOVA results show that this temperature influence on the effects of genetic variation on *r* is likely due to a combination of changes in the amount of expressed genetic variation in *r*, G, environmental changes in *r*, E, and strong G × E interactions (Table 1, Figure 2b). A post hoc test confirmed pairwise differences in growth rate among different clonal lines across temperatures (Table S1). Differences in *r* at 19ºC were mostly driven by clones CU438.1 and A*V, at 22ºC they were driven by differences between A*V and CU427.4 and B2086.2 as well as between B2086.2 and CU438.1, at 25ºC they were driven by all clonal lines growing faster than B2086.2, while no pairwise differences were observed at 28ºC (Table S1).

**TABLE 1:** (a) ANCOVA results assessing G, E, and G × E effects in *r*. (b) Effects of genetic background in *r*

	df	*F*‐statistic	*p*‐value
(a)			
G (differences across genotypes)			
At 19°C	3	6.56	.**003**
At 22°C	3	2.88	.062
At 25°C	3	34.6	**<10^−6^**
At 28°C	3	2.88	.059
E (differences across temperatures)			
Clone B2086.2	4	118	**<10^−8^**
Clone CU427.4	4	26.8	**<10^−5^**
Clone A*III	4	48.3	**<10^−7^**
Clone CU438.1	4	42.9	**<10^−6^**
Clone A*V	4	32.9	**<10^−6^**
G × E (differences across genotypes across temperatures)	12	4.70	**<10^−5^**
(b)			
Effect of genetic background			
Genetic Background	2	13.7	**<10^−4^**
Temperature	3	147	**<10^−29^**
Genetic Background*Temperature	6	6.37	**<10^−4^**

Note

Boldface indicates statistically significant result.

Differences in thermal responses across clonal lines were explained by differences in the genetic background of the different clonal species (Table 1b, Figure 2c). Indeed, lines from genetic backgrounds A, B, and C not only grew at different rates regardless of temperature (Table 1b) but also did so differently at different temperatures (significant Background*Temperature interaction, Table 1b), suggesting differential expression of those genetic backgrounds at different temperatures (Figure 2c). Indeed, at 19ºC and 22ºC, variation in *r* was mostly due to differences between clones with genetic backgrounds A and C, while at 25ºC there were no differences in *r* among clones of different genetic backgrounds (Table S2).

## 4 DISCUSSION

Rapidly changing environmental conditions and genetic diversity are both well‐known to independently influence population growth and extinction risk (Cooper et al., 2019; Freeman et al., 2018; Pimm, 2009; Weeks et al., 2017). Whether rapid climate change may alter how genetic diversity influences population growth, however, is not known. Our results indicate that as temperature increases toward a species’ thermal optimum, genetic diversity has a weaker effect on the intrinsic population growth rate (Figure 2a). These results imply that the effect of genetic diversity on population growth is contingent on both environmental conditions and physiological constraints on population growth.

While increasing genetic diversity resulting in higher population growth is common in other systems (Frankham, 2005), it is not clear why that should be the case in this particular study system. Previous work has indicated that inbreeding depression—that is, a decrease in absolute fitness (or intrinsic growth rate) with increasing levels of inbreeding (often due to the accumulation of deleterious alleles)—is unlikely to happen in *T*. *thermophila* (Dimond & Zufall, 2016), and outbreeding depression—that is, the decrease in absolute fitness due to the arrival of maladapted alleles—is also unlikely (Dimond & Zufall, 2016). Yet, our results very clearly indicate a strong increase in growth rates with genetic diversity that weakens at warmer temperatures (Figure 2a). One possible explanation for the observed increase in population growth with genetic diversity is the occurrence of heterosis, or outbreeding enhancement, which has not been ruled out in this particular system (Dimond & Zufall, 2016). Indeed, clonal lines from different genetic backgrounds may also belong to different mating types, which could have led to increasing levels of sexual reproduction and heterozygosity in genetically diverse combinations. However, we lack conclusive evidence of heterosis being at the basis of the observed increase in population growth and more research is needed to elucidate the precise mechanism through which that may happen in *T*. *thermophila*.

Our results also suggest that changes in expressed additive genetic variation in *r* (G) are at least in part responsible for the lower levels of variation in *r* observed at higher temperatures, compared to those observed at lower temperatures (Figure 2b, Table 1). On the other hand, plasticity (E) seemed responsible for the observed increase in population growth with temperature (Figure 2b). Moreover, strong G × E effects, where different genotypes grow differentially at different temperatures, likely underlie the weakening of the positive effect of genetic variation on population growth rate (Figure 2a): clonal lines grow at similar rates at warmer temperatures but do so at distinctly higher or lower rates in colder temperatures (Figure 2b).

Many of the observed differences in thermal response across clonal lines ultimately responsible for the observed levels of additive genetic variation (G) in *r*, and G × E interactions, may be due to differences in the genetic background of the different clonal lines (Table 1b, Figure 2c). Indeed, backgrounds A and C grew at similar rates at temperatures above 22ºC while background B grew much more slowly (Table 1b, Figure 2c), while clones from all three backgrounds grew at similar rates at 28ºC (Table 1b, Figure 2c). Moreover, while background A expressed high growth rates across all temperature ranges, C grew very slowly at 19ºC and B grew much faster at 28ºC, which explain the observed G × E interactions in *r* across temperatures.

Because the presence of strong G × E effectively shifts which genotypes grow better at different temperatures, there is a possibility for temperature‐mediated clonal sorting in these microbial populations. Rapid evolutionary change has been suggested as a possible mechanism through which organisms may fend off the negative impacts of climate change (Fox et al., 2019; Franks & Hoffmann, 2012; Franks et al., 2007; Geerts et al., 2015). In line with these studies, our results suggest that rapid evolutionary change (in this case, through clonal sorting) may occur in species where different genotypes display different thermal responses (G × E). However, we do not keep track of changes in allele frequencies in this study, so we do not know whether clonal sorting is happening differentially at different temperatures or at all, but this certainly is a promising avenue for future research.

We notice that the thermal performance curve of the species was quantified in a diverse population containing all five *T*. *thermophila* clones. While our experiment clearly indicates that the different clones differ in some aspects of their thermal responses (Figure 2b, c), it is unclear whether they differ in their *T*_opt_ or not. Two or more clones may in fact differ in their thermal responses in myriad ways. For example, they may differ in the shape of their TPC but not in their *T*_opt_, they may vary in their *T*_opt_ but not in the overall shape of their TPC, and they may vary in all aspects of the TPC, including *T*_opt_ (DeLong et al., 2018). In case the clonal lines did vary in their *T*_opt_, we would not have expected *r* to level with temperature (or only weakly) as was hypothesized in the introduction and then confirmed empirically (Figure 2b, c). This result therefore suggests that the clonal lines differ in the shape of their TPCs, but not *T*_opt_, perhaps due to strong physiological and thermodynamical constraints at play past *T*_opt_, as suggested elsewhere (Pawar et al., 2016). Alternatively, there could be rapid clonal sorting occurring in the diverse microcosms used to quantify the species‐level TPC, which would lead to a species‐level TPC that more closely reflect the behavior of specific clones at specific temperatures, rather than the actual TPC of the whole species. However, due to the short timeframe used to quantifying the TPC, we suspect that is not the case.

Importantly, our study does not address whether or how temperature may influence the effect of genetic variation on population growth on the declining portion of the TPC (i.e., past *T*_opt_), which represents a clear next step. We hypothesize that as temperature rises past *T*_opt_, we should see a strengthening of the effect of genetic diversity on *r* (i.e., as the distance between the treatment temperature and *T*_opt_ increases), for the same reasons that we observe a weakening of the effect of genetic diversity on *r* as temperature increases toward *T*_opt_. If that is the case, increasing genetic diversity could “rescue” populations (*sensu* Carlson et al., 2014) that would otherwise have negative growth at higher temperatures. This thermal genetic rescue effect represents an exciting new avenue for future research with both fundamental and applied consequences.

Together, our results indicate possible ways in which increasing temperatures associated with climate change and depauperate genetic stocks resulting from habitat fragmentation may jointly affect population growth and extinction risk. We show that temperature and genetic diversity interactively influence population growth: populations with higher genetic diversity have a weaker response to temperature compared to genetically depauperate populations (Figure 2a). As a consequence, while genetic diversity hedges against increasing temperatures, inbred—or small—populations may respond more strongly. These results have important implications for the management of threatened and other species of interest in a changing world.

## CONFLICTS OF INTEREST

None declared.

## AUTHOR CONTRIBUTIONS

**Alexandra Singleton:** Conceptualization (equal); Data curation (lead); Formal analysis (lead); Investigation (lead); Writing‐original draft (lead); Writing‐review & editing (lead). **Megan Liu:** Investigation (supporting). **Samantha Votzke:** Investigation (supporting); Writing‐review & editing (supporting). **Andrea Yammine:** Conceptualization (supporting); Investigation (supporting); Writing‐review & editing (supporting). **Jean P. Gibert:** Conceptualization (equal); Data curation (supporting); Formal analysis (supporting); Funding acquisition (lead); Resources (lead); Supervision (equal); Writing‐original draft (supporting); Writing‐review & editing (equal).

### OPEN RESEARCH BADGES

This article has been awarded <Open Data, Open Materials>Badges. All materials and data are publicly accessible via the Open Science Framework at https://doi.org/10.5061/dryad.sqv9s4n52; https://github.com/JPGibert/Genetic_Diversity_And_Temperature

## Supporting information

Appendix S1

## Data Availability

All data and code are publicly available in Dryad (https://doi.org/10.5061/dryad.sqv9s4n52) or at https://github.com/JPGibert/Genetic_Diversity_And_Temperature.
