# Supplementary material for: Increasing temperature weakens the positive effect of genetic diversity on population growth
Source: Ecol Evol. 2021 Dec 14;11(24):17810–6. doi: 10.1002/ece3.8335 (PMC8717318; doi:10.1002/ece3.8335)

Supplementary Materials: Increasing temperature weakens the positive effect of genetic diversity on population growth

Alexandra L. Singleton^1,*^, Samantha Votzke^1^, Andrea Yammine^1^, Jean P. Gibert^1,*^

^1^Duke University, Department of Biology, Durham, NC, USA

*To whom correspondence should be addressed: [alexsingleton2@gmail.com](mailto:alexsingelton2@gmail.com), [jean.gibert@duke.edu](mailto:jean.gibert@duke.edu)


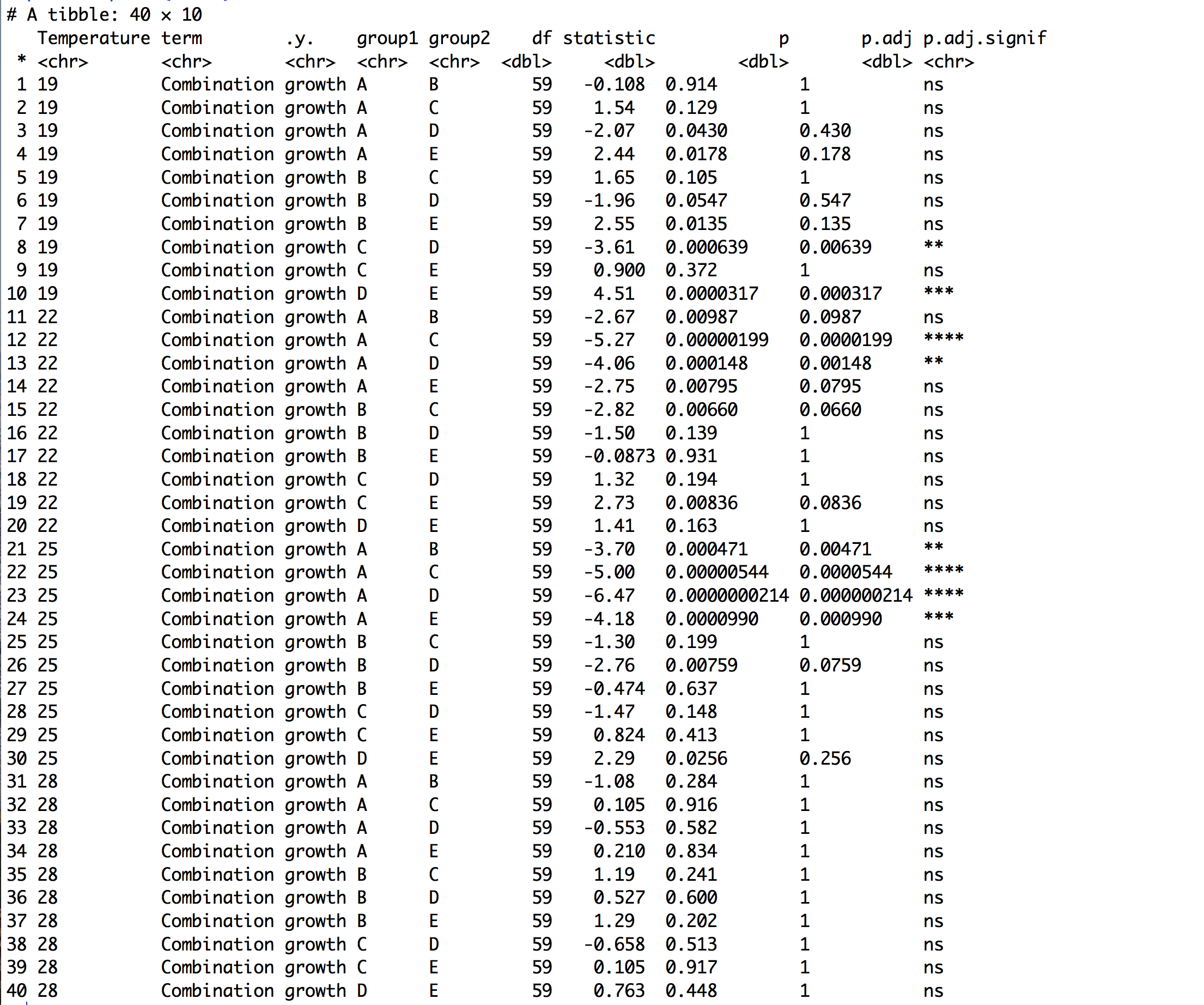
Table S1: Post-hoc test with Bonferroni-adjusted p-values for the pairwise comparisons in *r* among all clones and for each temperature. For the purpose of this analysis, clones are named as follows: B2086.2=A, A*III=B, CU438.1=C, A*V=D, CU427.4=E.

Table S2: Post-hoc test with Bonferroni-adjusted p-values for the pairwise comparisons in *r* among clones with different genetic backgrounds (A, B and C). Clones belonging to the different genetic backgrounds are: A=A*III and A*V, B= B2086.2, C= CU438.1 and CU427.4.


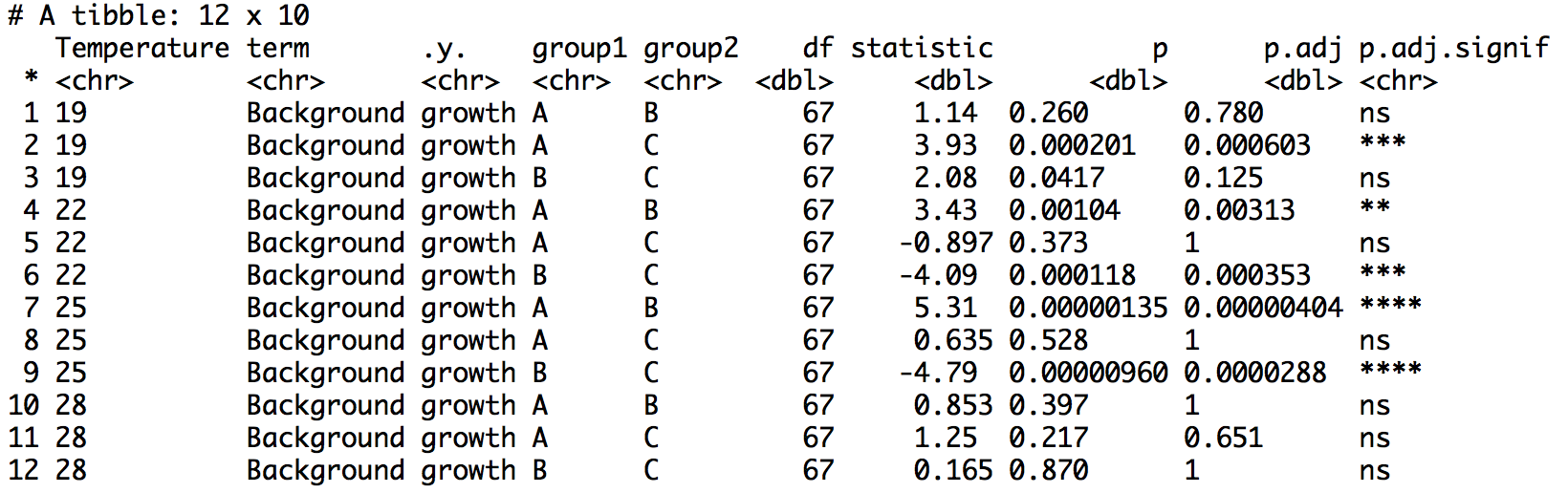

Supplement: Supplementary file 1 — Appendix S1 [file ECE3-11-17810-s001.docx]
